# Familial tetrasomy 4q35.2 associated with congenital diaphragmatic hernia and unilateral renal agenesis: a case report

**DOI:** 10.1186/s13256-016-0855-1

**Published:** 2016-03-30

**Authors:** Thomas Bogs, Florian Kipfmüller, Nicolai Kohlschmidt, Ulrich Gembruch, Andreas Müller, Heiko Reutter

**Affiliations:** 1 Department of Neonatology and Pediatric Intensive Care, Children’s Hospital University of Bonn, Sigmund-Freud-Strasse 25, D-53127 Bonn, Germany; 2 Institute of Clinical Genetics, Bonn, Germany; 3 Department of Obstetrics and Prenatal Medicine, University of Bonn, Bonn, Germany; 4 Institute of Human Genetics, University of Bonn, Sigmund-Freud-Strasse 25, D-53127 Bonn, Germany

**Keywords:** Case report, Congenital diaphragmatic hernia, Unilateral renal agenesis, Ear anomaly, Partial tetrasomy 4q35.2

## Abstract

**Background:**

Previous reports of chromosomal aberrations in different forms of congenital diaphragmatic hernia have been described as comprising aneuploidies (for example, trisomy 21), microdeletions, and duplications (for example, monosomy 15q24, 22q11.2).

**Case presentation:**

We describe the first association of a *de novo* partial tetrasomy 4q35.2 in a father with left-sided, isolated renal agenesis and left-sided, isolated congenital diaphragmatic hernia in his son, who inherited the chromosomal aberration from his father.

**Conclusions:**

Given that the aberration occurred *de novo* in the father and was transmitted to his son, with both presenting with unilateral left-sided developmental field defects, we suggest a gene dosage effect of the tetrasomic region to be involved in the phenotype of our two patients. Furthermore, we suggest performing a genetic workup in multiplex families with congenital malformations.

## Background

Congenital diaphragmatic hernia (CDH) represents a spectrum of rare developmental defects resulting from an aberrant embryonic morphogenesis of the developing diaphragm. The diaphragmatic defect allows an upward displacement of the abdominal organs into the thorax with a consecutive compression of the intrathoracic compartment, leading to hypoplasia of the lung, secondary pulmonary hypertension, and intestinal malrotation [1]. CDH occurs with a birth prevalence of 1 in 3000–4000 [2]. Long-term complications are gastroesophageal reflux disease, failure to thrive, neurocognitive defects, and hearing loss [3, 4]. In approximately 50–60 % of patients, CDH occurs as isolated malformation [2, 5]; about 40 % present with additional anomalies of other organ systems, including the central nervous system (5–75 %), the cardiovascular system (4–63 %), the genitourinary system (5–27 %), and the gastrointestinal tract (1–20 %) [6]. Among the associated genitourinary anomalies, renal anomalies are common, comprising renal agenesis, dysplasia, hypoplasia, or hydronephrosis [7]. In the past, conventional karyotyping identified chromosomal anomalies in about 6.3 % of cases [2]; in recent studies, researchers used microarray analysis and identified smaller genomic loci, with and without candidate genes [1, 8]. However, the etiology of CDH is still unknown in more than 70 % of patients [2]. In this report, we present the first case of a *de novo* partial tetrasomy 4q35.2 in a father with isolated unilateral renal agenesis and isolated CDH in his son, respectively.

## Case presentation

The index patient is the first child of his nonconsanguineous parents. Due to reduced fertility of the father, assisted reproduction in the form of intracytoplasmic sperm injection had been performed to achieve pregnancy in the couple. Besides his reduced fertility, the father was born with unilateral agenesis of the left kidney and a poorly formed hypoplastic helix of his left ear. He was a university graduate and was working in a management position.

**Fig. 1:**
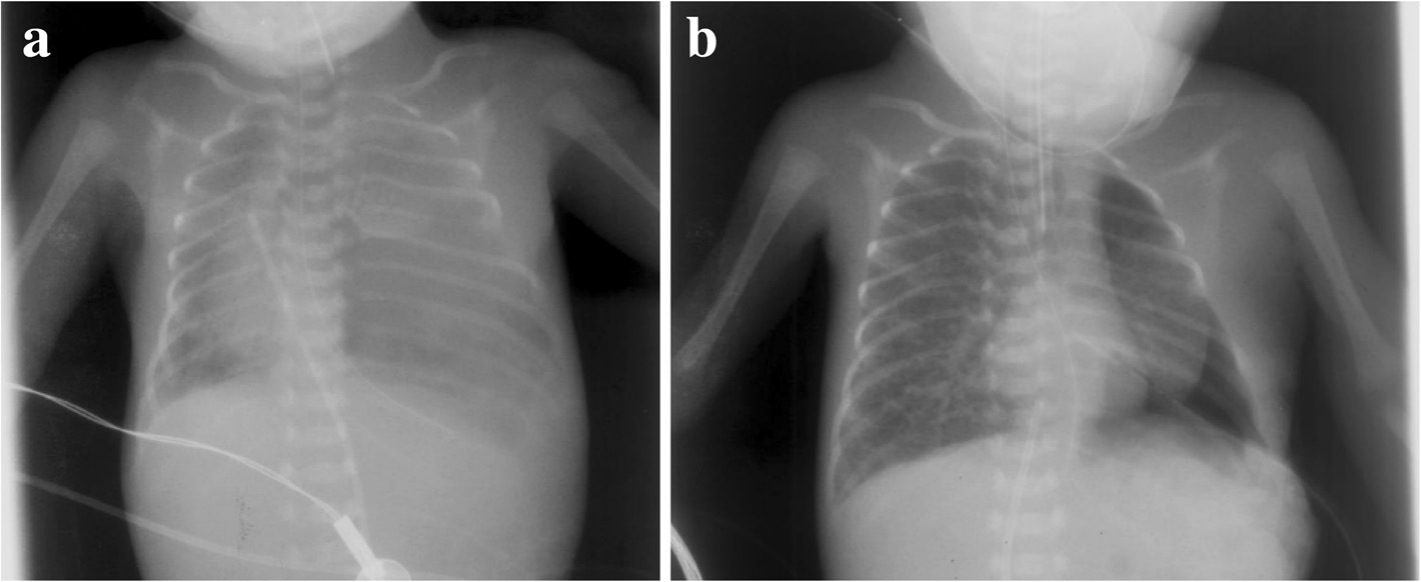
Preoperative (**a**) and postoperative (**b**) x-rays of the index patient

The achieved pregnancy was uneventful until the second trimester, when prenatal ultrasound of the fetus revealed isolated left-sided CDH. The mother gave birth to a boy at 27 + 2 weeks of gestation by cesarean section due to preterm rupture of the membranes and contractions despite intravenous tocolysis. The boy’s birth weight was 950 g (25th percentile), length was 36.6 cm (50th percentile), and head circumference was 25 cm (30th percentile). His Apgar scores were 6 at 1 minute, 8 at 5 minutes, and 8 at 10 minutes. He was intubated immediately after delivery and ventilated. A chest X-ray confirmed left-sided CDH (Fig. 1a). After his circulation and respiration stabilized, the boy underwent surgical correction of the CDH on the 11th day of life (Fig. 1b). He was successfully weaned off mechanical ventilation on the 26th day of life, and oxygen supplementation could be seized on day 61 of life. The neonatal period was complicated by an intraventricular hemorrhage with consecutive dilation of the ventricles, which was eventually treated by implantation of a Rickham reservoir on the 28th day of life. A ventriculoperitoneal shunt was implemented 4 months after birth. By the time of hospital discharge, the boy presented with general developmental delay. However, because he was born prematurely and had periods of severe hypoxia due to oxygenation difficulties due to his CDH condition, and because his neonatal period was further complicated by an intraventricular hemorrhage, it was impossible to distinguish whether his developmental delay was primary or secondary.

**Fig. 2:**
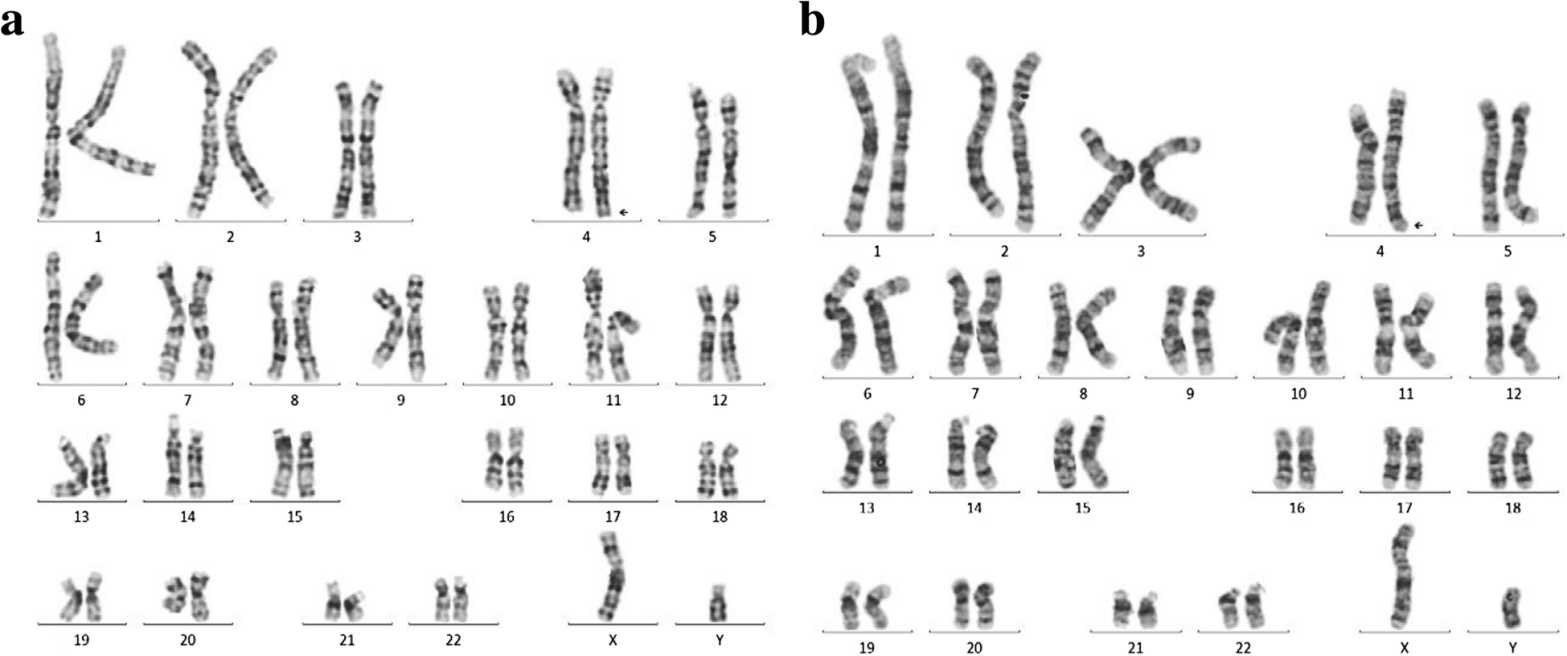
GTG banding of amniotic fluid cells in the fetus (**a**) and of peripheral blood lymphocytes from the father (**b**) revealed a triplication of chromosomal region 4q35.2 spanning approximately 3.73–4.43 Mb ([46,XY,qdp{4}{q35.2qter}]) (see *arrows*)

During the pregnancy of the index patient, amniocentesis and conventional karyotyping based on GTG staining at 450 bands per haploid genome level at 16 + 5 weeks of gestation was suggestive of partial tetrasomy 4q35.2 in the male fetus (Fig. 2a). For further characterization, fluorescence *in situ* hybridization (FISH) analysis was performed, which revealed an increased signal at 4qter only. Multiple ligation-dependent probe amplification (genomic region chr4:185,963,614–189,037,586, kits P036-E1, P070-B1, and P264-A1; MRC-Holland, Amsterdam, the Netherlands) of the specific region confirmed a triplication of chromosomal region 4q35.2 spanning approximately 3.73–4.43 Mb ([46,XY,qdp{4}{q35.2qter}]) in one of the two chromosomes 4 of the fetus. To test whether the chromosomal aberration occurred *de novo*, and because the father presented with reduced fertility and unilateral renal and helix anomaly, consecutive karyotyping of the parents was performed, which revealed the same aberration in the father (Fig. 2b). Cytogenetic analysis of the mother was unremarkable, as was the FISH analysis of the subtelomeric regions of both her chromosomes 4.

Genetic counseling of the parents during the hospital course of their son prompted them to ask the paternal grandparents of the index patient to have their karyotypes analyzed to see if the triplication of chromosomal region 4q35.2 had occurred *de novo* in the father. Conventional karyotyping of the paternal grandparents showed normal results, confirming that the partial tetrasomy of chromosomal region 4q35.2 in the father had occurred *de novo*.

## Discussion

In this report, we present the first case of partial tetrasomy 4q35.2 [(46,XY,qdp{4}{q35.2qter})] and associated CDH, unilateral renal agenesis, and ear anomaly in the carriers. Previous reports of chromosomal aberrations in different forms of CDH described aneuploidies (for example, trisomy 21), microdeletions and duplications (for example, monosomy 15q24, 22q11.2) [6, 8, 9]. Approximately 10 % of patients with CDH have it in the context of monogenic syndromes such as Fryns syndrome [6]. While CDH usually occurs sporadically, reports of familial cases are suggestive of an underlying Mendelian mode of inheritance in some of the families [10, 11]. In accordance with this, patients with CDH and single-gene mutations in *FOG2* [12], *GATA4* [13], and *FREM1* [14] have been reported.

Regarding the aberrant region on 4q35.2 that we describe in this report, we conducted a medical database search using PubMed (www.ncbi.nlm.nih.gov/pubmed) and retrieved several reports of patients with overlapping deletions. Some of these patients were described to have birth defects [15, 16]. However, we retrieved far fewer reports of overlapping duplications. Among these, Lin *et al.* [17] described a patient with congenital bilateral choanal atresia and several other phenotypic anomalies. Interestingly, we retrieved 94 entries with overlapping duplications of chromosomal region 4q35.2 from the DECIPHER database v9.0 in September 2015 (Database of Chromosomal Imbalance and Phenotype in Humans; https://decipher.sanger.ac.uk/). One of these was a female patient [accession number 4647] presenting with, among other anomalies, unilateral renal agenesis, low-set ears, and ventricular septal defect. Furthermore, a male patient [accession number 278863] presented with, among other anomalies, CDH and posteriorly rotated ears. The phenotypic variation between the father and the son described in the present report might be due to different genetic mutations in the genes downstream of the triplicated genes or that other genes not involved in the triplication may compensate for or inhibit the pathogenesis of the triplication to different degrees [18]. Hence, triplication of the region may have induced the CDH and renal agenesis indirectly by promoting or silencing genes lying downstream. It is also possible that a direct dosage effect of the comprised genes (for example, *FAT1*) lead to CDH and renal agenesis. *FAT1* (NM_005245) encodes a tumor suppressor essential for controlling cell proliferation during *Drosophila* development. According to the Mouse Genome Informatics database (http://www.informatics.jax.org/), homozygous, targeted null mutations exhibit holoprosencephaly, anophthalmia, kidney defects, and perinatal lethality.

## Conclusions

Overall, our observations suggest that duplication or triplication of chromosomal region 4q35.2 carries susceptibility for the development of renal, ear, and diaphragmatic anomalies. Furthermore, we suggest performing a genetic workup of multiplex families with congenital malformations.

## Consent

Written informed consent was obtained from the patient’s legal guardian for publication of this case report and any accompanying images. A copy of the written consent is available for review by the Editor-in-Chief of this journal.

## Abbreviations

CDH: congenital diaphragmatic hernia
FISH: fluorescence *in situ* hybridization
